# Inter/intra-frame constrained vascular segmentation in X-ray angiographic image sequence

**DOI:** 10.1186/s12911-019-0966-x

**Published:** 2019-12-19

**Authors:** Shuang Song, Chenbing Du, Ying Chen, Danni Ai, Hong Song, Yong Huang, Yongtian Wang, Jian Yang

**Affiliations:** 1 Beijing Engineering Research Center of Mixed Reality and Advanced Display, School of Optics and Photonics, Beijing Institute of Technology, Beijing, 100081 China; 2 AICFVE of Beijing Film Academy, 4 Xitucheng Rd, Haidian, Beijing, 100088 China; 3 School of Computer Science & Technology, Beijing Institute of Technology, Beijing, 100081 China

**Keywords:** X-ray angiographic image sequence, Vascular enhancement, Multi-feature, Vascular segmentation

## Abstract

**Background:**

Automatic vascular segmentation in X-ray angiographic image sequence is of crucial interest, for instance, for better quantifying coronary arteries in diagnostic and interventional procedures.

**Methods:**

A novel inter/intra-frame constrained vascular segmentation method is proposed to automatically segment vessels in coronary X-ray angiographic image sequence. First, a morphological filter operator is applied to remove structures undergoing the respiratory motion from the original image sequence. Second, an inter-frame constrained robust principal component analysis (RPCA) is utilized to remove the quasi-static structures from the image sequence. Third, an intra-frame constrained RPCA is employed to smooth the final extracted vascular sequence. Fourth, a multi-feature fusion is designed to improve the vascular contrast and the final vascular segmentation is realized by thresholding-based method.

**Results:**

Experiments are conducted on 22 clinical X-ray angiographic image sequences. The global and local contrast-to-noise ratio of the proposed method are 6.6344 and 4.2882, respectively. And the precision, sensitivity and F1 value are 0.7378, 0.7960 and 0.7658, respectively. It demonstrates that our method is effective and robust for vascular segmentation from image sequence.

**Conclusions:**

The proposed method is effective to remove non-vascular structures, reduce motion artefacts and other non-uniform illumination caused noises. Also, the proposed method is online which can just process one image per time without re-optimizing the model.

## Background

Nowadays, coronary artery disease (CAD) is greatly threatening human health [1]. Since X-ray angiography (XRA) has better imaging quality and faster imaging speed, it is regarded as the gold standard for the diagnosis and treatment of CAD. However, due to the perspective projection of 3D anatomic structures, much 3D information has been lost and different anatomical structures are overlapped in the XRA images. Moreover, the injection of contrast agent and the blood flow variation bring in-homogeneous intensity of coronary artery. And the cardiorespiratory motion and patient movement also introduce motion artefacts to the XRA images. To improve the image-guided diagnosis and interventional procedures of CAD, the automatic and robust vessel segmentation is of great significance and meanwhile a challenging problem.

Vascular segmentation technique can be divided into two classes, including the model-based and learning-based methods. Based on the spatial continuity of vessels, level-set and active contour-based methods are commonly utilized. Wang et al. [2] utilized the level-set algorithm to segment the coronary artery by constructing the speed function with the curvature, intensity and model term. Sun et al. [3] proposed the local region based active contour method by shape fitting the energy function. It improved the segmentation accuracy and is much more robust to the non-uniform intensity distribution and fitting initialization. Based on the shape of vascular section, Cheng et al. [4] employed a B-snake model to accurately segment the small-scale vessels in the low-contrast images. Lee et al. [5] utilized the Kalman filter to initialize the contour and segmented the vessels by the active contour model. The initialization improved the time efficiency. The learning-based methods usually compute the classification model based on the image hidden information. Hassouna et al. [6] modeled the background with two Gaussian and a rayleigh distributions and the vessels with a Gaussian distribution, respectively. Then they utilized the Expectation-Maximization algorithm to estimate the distribution parameters and employed the Markov Random Field to be the spatial constraint to realize the final vascular segmentation. Goceri et al. [7] clustered the vessels based on the K-means approach and improved the segmentation accuracy by the morphology based iterative optimization. Lupascu et al. [8] delivered the high order features to the AdaBoost classifier to speed the segmentation. Orlando et al. [9, 10] computed the fused feature map by the Fully-Connected Conditional Random Field to ensure the continuity of different vascular segments. In recent years, Convolutional Neural Network (CNN) based vascular segmentation has attracted much researcher’s attention. Wang et al. extracted the vascular features based on CNN to segment the vessels with a stochastic decision forest. Fu et al. [11] combined CNN with Conditional Random Field (CRF) and developed a DeepVessel network to improve the segmentation accuracy. Luo et al. [12] improved the DeepVessel network by considering the non-uniform intensity and noise coexistence.

To the authors’ knowledge, the model-based segmentation methods are sensitive to the initial contour and learning-based approaches require large amounts of labeled datasets. Moreover, the methods mentioned above have a significant limitation in angiographic images with low contrast and noisy background. Vascular enhancement can greatly ease the vascular segmentation by enhancing the vascular structures and compress the background noise. The single-image based enhancement easily introduces the non-vascular noise and motion artefacts when dealing with X-ray angiogram images. While subtraction-based enhancement utilizes the angiograms with and without vessels. It can effectively remove the motion artefacts in the final enhanced vascular angiograms and improve the subsequent segmentation accuracy.

Current vessel subtraction methods can be classified into two categories, including image registration based methods and layer separation based methods. In the imaging of coronary artery, mask images are taken prior to the perfusion of the contrast agent and coronary arteries are not visible in them. While live images are taken during the contrast agent passing through the coronary artery. Image registration based methods [13, 14] only need a live image and a mask image whose motion is the most similar to the live image. Such methods are usually realized by template matching, similarity measure maximization, image warping and subtraction technology sequentially. Though the technique largely reduces the motion artifacts and non-vascular noise, it is likely to be interrupted by the patient motion or contrast agent leakage when computing the correspondences between images. Motion layer separation based method supposes an image in the sequence can be decomposed into motion layers. The key part of the first type methods [15–17] is motion estimation of each layer. Zhu et al. [15] divided the sequence into the vascular and non-vascular layers and applied optical flow to the non-vascular layer to compute the deformation filed. Zhang et al. [16] separated the sequence into three layers, including static, lung (slow motion) and vessel (rapid motion) layer and constructed a motion transformation model for each layer. Nevertheless, the structures in the XRA sequence participate in different motion patterns. Specific motion model in each layers cannot cover all the motions of a structure, especially vascular motion in the XRA sequences includes the cardiac, respiratory, patient and camera motions. Another kind of methods [18–20] supposes that the image is under specific prior constraint and directly separates the sequence into background and vessel layer. Many mathematical expressions, such as *L1* norm, *L2* norm, nuclear norm and so on, have been applied to model the specific prior. Robust principal component analysis (RPCA) model, composed of sparse and low-rank prior, has become a common tool in medical image analysis of various imaging modalities.

In this paper, we propose an inter/intra-frame constrained vascular segmentation in the angiographic image sequence. First, a morphological filter operator is applied to remove motion artefacts caused by respiratory motion from original XRA sequence. Second, an inter/intra-frame constrained RPCA (IFC-RPCA) is utilized to extract the vascular images. Third, a multi-feature fusion is designed to realize the final vascular segmentation. The proposed method is effective to remove non-vascular structures, reduce motion artefacts, other non-uniform illumination caused noise and preserve the local information of vascular structures.

## Methods

In this section, the vascular images is distinguished from the XRA sequence by removing the structures that are static or undergoes the respiratory motion. Then, a multi-feature fused descriptor is employed to further compress the background noise in the vascular images and the vascular structures are finally segmented by a thresholding-based approach.

### Inter/intra-frame constrained RPCA

To extract the vascular images from the XRA sequence *I*, structures that are static or undergo the respiratory motion should be removed. To reduce the disturbance (lung, diaphragm) caused by the respiratory motion, a circular structural element based morphological close operation [18] is applied to the sequence *I* to obtain the respiratory sequence *R*. By subtracting the sequence *R* from sequence *I*, the respiratory disturbance can be removed and the obtained sequence is denoted as *DI*. The sequence *DI* is composed of two components, including the moving vascular component and the quasi-static non-vascular component. In addition, the vessels in sequence *DI* only occupy a small portion. Considering RPCA aims to decompose the matrix into a low-rank component and an overall sparse component by searching for a low-dimensional subspace, it is suitable to separate sequence *DI* into the moving vascular component and the quasi-static non-vascular component [21]. Hence, we have:

1 $$ \left\{B,E\right\}=\arg \min \frac{1}{2}{\left\Vert DI-B-E\right\Vert}_F^2+{\beta}_1{\left\Vert B\right\Vert}_{\ast }+{\beta}_2{\left\Vert E\right\Vert}_1 $$

where *E* refers to the vascular component, and *B* is the quasi-static non-vascular component. *β*_1_ and *β*_2_ are regularization coefficients. ‖∙‖_*F*_ is the Frobenius norm, ‖∙‖_∗_ is the nuclear norm and ‖∙‖_1_ is L1 norm.

Considering dealing with the steaming X-ray images for coronary interventions, the online processing of XRA sequence is essential on the basis of the motion information in inter-frames. Hence, we utilize the explicit low-rank factorization [21] to describe *B* by the subspace basis *Lr* and the corresponding coefficients *Ce*. The factorization can be denoted as *B* = *Lr* × *Ce*^*T*^. After this, solving Eq. (1) equals to minimize the empirical cost function *g*(*Ce*, *E*, *Lr*), and we have:

2 $$ g\left( Ce,E, Lr\right)=\frac{\lambda_1}{2\times N}{\left\Vert Lr\right\Vert}_F^2+\frac{1}{N}\sum \limits_{i=1}^N\left({\left\Vert D{I}_i-L{r}_{\mathrm{i}}C{e}_i^T-{E}_i\right\Vert}_2^2+\frac{\lambda_1}{2}{\left\Vert C{e}_i\right\Vert}_2^2+{\lambda}_2{\left\Vert E\right\Vert}_1\right) $$

where *DI*_*i*_ is the *ith* image of sequence *DI*, *N* is the number of images in sequence *DI*. $L{r}_i\in {\mathcal{R}}^{D\times r}$, $C{e}_i\in {\mathcal{R}}^r$, *D* is the dimension of an image in sequence *DI*, *r* is the upper bounded rank of *B*. In the optimization of Eq. (2), coefficients *Ce*, vascular component *E* and basis *Lr* are disposed in an alternative manner. In the alternative manner, {*Ce*_*i*_, *E*_*i*_} of the *ith* image in *DI* is computed by the *ith* image and *Lr*_*i* − 1_ of the (*i-1) th* image. Then, *Lr*_*i*_ of the *ith* image is re-computed on the basis of {*Ce*_*i*_, *E*_*i*_}. By repeating the procedure, vascular component in each image of sequence *DI* can be computed by combining the motion information of vascular structures as an inter-frame constraint. After the inter-frame constrained RPCA, the vascular component is separated from the quasi-static non-vascular component in the XRA sequence.

However, due to the non-rigid motion between the frames in the XRA sequence, large amount of motion artefacts and noises may still exist in the vascular component. Hence, we utilize the same morphological close operation to remove the motion artefacts around the catheter and obtain another difference sequence *DI*^′^. To remove more motion artefacts and noises, we introduce the intra-frame constrained RPCA and denote it according to Eq. (2) as follows:

3 $$ g\left(C{e}^{\prime },V,L{r}^{\prime}\right)=\frac{1}{N}{\sum}_{i=1}^N\left({\left\Vert D{I}_i^{\prime }-L{r}_i^{\prime }C{e}_i^{\prime T}-{V}_i\right\Vert}_2^2\right)+\frac{\lambda_1}{2}{\left\Vert C{e}_i^{\prime}\right\Vert}_2^2+{\lambda}_2{\left\Vert {V}_i\right\Vert}_1+\frac{\lambda_1}{2\times N}{\left\Vert L{r}^{\prime}\right\Vert}_F^2 $$

where $L{r}_i^{\prime}\in {\mathcal{R}}^{D\times {r}^{\prime }}$, $C{e}_i^{\prime}\in {\mathcal{R}}^{r^{\prime }}$. In addition, since most static structures have been removed in the inter-frame constrained RPCA, the optimization of intra-frame constrained RPCA will not depend on the motion information across the image sequence *DI*^′^. Hence, we utilize a **1** matrix as $L{r}_i^{\prime }$ and the optimization of intra-frame constrained RPCA only need to update $\left\{C{e}_i^{\prime },{E}_i^{\prime}\right\}$, as follows:

4 $$ \left\{C{e}_i^{\prime },{V}_i\right\}=\mathit{\arg}\mathit{\min}\frac{1}{2}{\left\Vert D{I}_i^{\prime }-L{r}_i^{\prime }C{e}_i^{\prime }-{V}_i\right\Vert}_2^2+\frac{\lambda_1^{\prime }}{2}{\left\Vert C{e}_i^{\prime}\right\Vert}_2^2+{\lambda}_2^{\prime }{\left\Vert {V}_i\right\Vert}_1 $$

Through the intra-frame constrained RPCA, the final enhanced vascular sequence *V* is obtained. In sequence *V*, the contrast of vessels in the images is improved and the background is smooth and clean.

### Multi-feature fused vascular segmentation

For each image *V*_*i*_ in *V*, we utilize *VI*_*i*_(*x*) to represent the intensity of *i th* image in *VI* at *x*, and *x* = [*x*_1_, *x*_2_]^*T*^ which refers to the pixel coordinate. The Hessian matrix at scale *σ* can be computed as follows:

5 $$ H\left(x,\sigma \right)={\sigma}^2{VI}_i(x)\ast \frac{\partial^2}{\partial {x}_1\partial {x}_2}\frac{\mathit{\exp}\left(-{x}^Tx/2{\sigma}^2\right)}{2\pi {\sigma}^2} $$

We use *λ*_1_ and *λ*_2_ to be the eigenvalues of matrix *H*, and *v*_1_ and *v*_2_ to be the corresponding eigenvectors of matrix *H*. For the pixels belong to the vascular structures, the eigenvalues should satisfy the principle |*λ*_1_| ≈ 0, |*λ*_1_| ≪ |*λ*_2_|. The directions of eigenvectors *v*_1_ and *v*_2_ are along with the vascular centerline and perpendicular to the vascular tangential direction, respectively.

Since the vascular structures contain the elongate and round-sectional segments (bending, bifurcations and diseased vascular segments), a good vascular feature descriptor should distinguish the vascular segments with other structures. Hence, we design a new vascular feature descriptor. To avoid the compression of feature descriptor, the first feature descriptor [22] *F*_1_(*x*, *σ*) at each pixel is computed as follows:

6 $$ {F}_1\left(x,\sigma \right)=\left\{\begin{array}{cc}\mathit{\ln}\left({\lambda}_2^2\left(x,\sigma \right)+1\right)& {\lambda}_2\left(x,\sigma \right)<-\sqrt{2\pi}\sigma \\ {}0& else\end{array}\right. $$

However, *F*_1_(*x*, *σ*) has a non-uniform response when the bending and bifurcation of vascular segments appear and is easily changed by the non-uniform illumination introduced by the contrast agent infusion. To avoid these conditions, another feature descriptor [23] *F*_2_(*x*, *σ*) at each pixel is calculated as follows:

7 $$ {F}_2\left(x,\sigma \right)=\left\{\begin{array}{cc}0& {\lambda}_2\left(x,\sigma \right)\le 0,{\lambda}_r\left(x,\sigma \right)\le 0\ \\ {}1& {\lambda}_2\left(x,\sigma \right)\ge \frac{\lambda_r\left(x,\sigma \right)}{2}>0\\ {}{\lambda}_2^2\left(x,\sigma \right)\left({\lambda}_r\left(x,\sigma \right)-{\lambda}_2\left(x,\sigma \right)\right){\left(\frac{3}{\lambda_r\left(x,\sigma \right)+{\lambda}_2\left(x,\sigma \right)}\right)}^3& else\end{array}\right. $$

And,

8 $$ {\lambda}_r\left(x,\sigma \right)=\left\{\begin{array}{cc}{\lambda}_2\left(x,\sigma \right)& {\lambda}_2\left(x,\sigma \right)>\underset{x}{\max }{\lambda}_2\left(x,\sigma \right)\ \\ {}\underset{x}{\max }{\lambda}_2\left(x,\sigma \right)& 0<{\lambda}_2\left(x,\sigma \right)\le \underset{x}{\max }{\lambda}_2\left(x,\sigma \right)\\ {}0& else\end{array}\right. $$

But *F*_2_(*x*, *σ*) appears serious blurring when the vascular segments are overlapped or very close to each other. Hence, to improve the vascular contrast and compress the non-vascular structures, we fuse the two feature descriptor with a weighted pattern to produce the uniform response of vascular segments and improve the boundary accuracy of vascular segments:

9 $$ F(x)=\underset{\sigma_{min}\le \sigma \le {\sigma}_{max}}{\max}\left({\alpha}_1{F}_1\left(x,\sigma \right)+{\alpha}_2{F}_2\left(x,\sigma \right)\right) $$

Until now, we obtain a feature value for each pixel in image *VI*_*i*_. Since the new feature descriptor can effectively distinguish the vascular and non-vascular pixels, the segmented vascular structure image *SI*_*i*_ can then be obtained from image *VI*_*i*_ by only utilizing a threshold value.

## Experimental results

All the experiments were implemented in MATLAB (The MathWorks, Inc.) under the Windows 10 environment, and all the experiments were conducted on a relatively low-cost PC with 16 GB RAM and 3.2 GHz Intel CPU.

### Dataset and evaluation criteria

The proposed method was evaluated on 22 XRA sequences collected from the Peking Union Medical College Hospital. The size of all the images in the sequences is 512 × 512 and the resolution of each image is 0.3 × 0.3 mm^2^. In all 22 XRA sequences, the inflow and wash out of contrast agent in the whole coronary artery are all recorded during the imaging procedure.

**Fig. 1:**
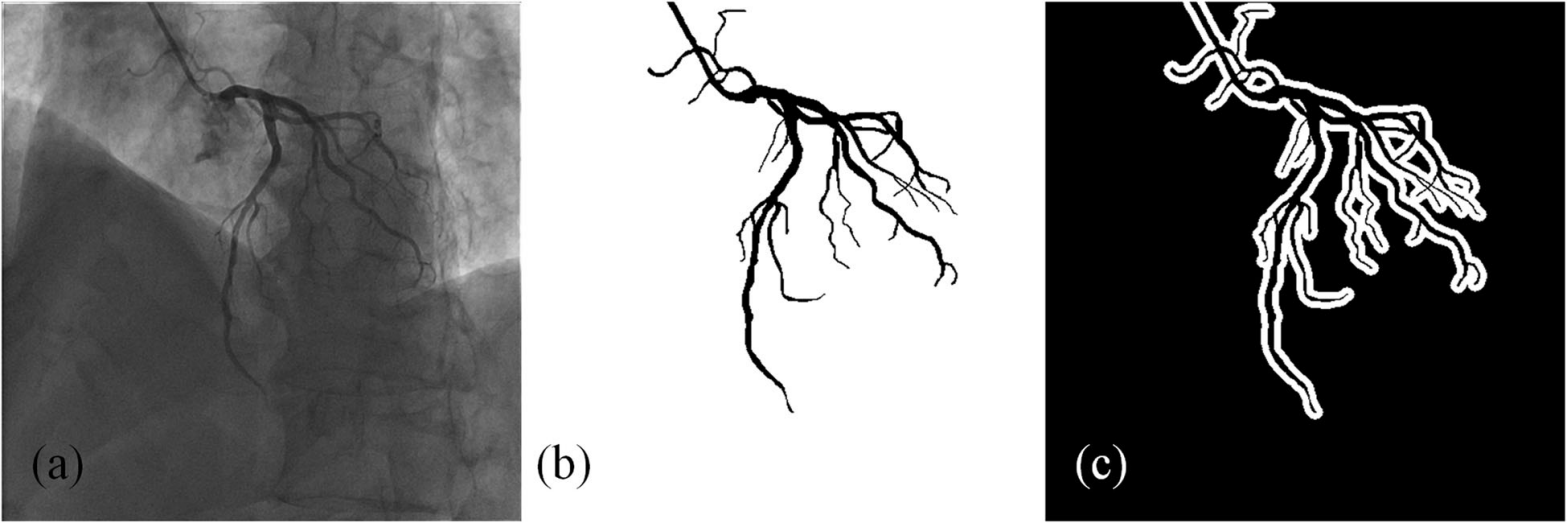
An example of masks that are utilized for quantitatively validation of the proposed method. **a** the original image; **b** global mask; **c** local mask

To quantitatively evaluate the proposed IFC-RPCA method, two kinds of masks are generated. One is used to evaluate the global vascular contrast and pixels within and outside the annotated vascular region consist of the vessels and background, as shown in Fig. 1b. The other one is used to evaluate the local vascular contrast. As shown in Fig. 1c, vascular region is the same with the global mask, and the background region is comprised of the pixels in the white region, which is the 7-pixel-wide neighborhood of the vascular region boundary.

To evaluate the performance of the proposed method, it will be compared with the multiresolution elastic registration (MER) method [14], the online robust principal component analysis (ORPCA) method [19], the graduated RPCA with motion coherency constraint (MCR-RPCA) method [20]. To evaluate the proposed segmentation method, it will be compared with Fully-Connected Conditional Random Field (FC-CRF) method [10] and level-set-based method (LevelSet) [24].

Contrast-to-noise ratio (CNR) [18] is utilized to evaluate the vascular contrast of the vessels and can be defined as follows:

10 $$ CNR=\frac{\left|{\mu}_F-{\mu}_B\right|}{\sigma_B} $$

where *μ*_*F*_ and *μ*_*B*_ are the mean gray values in the vascular and background regions in the extracted vascular images. *σ*_*B*_ is the standard deviation of the gray values in the background region. We compute the global CNR and local CNR based on global mask and local mask, respectively.

To evaluate the proposed segmentation method, we utilize five metrics including the precision (*pre*), sensitivity (*sen*) and F1 value. In addition, the metrics are computed as follows:

11 $$ \left\{\begin{array}{c} pre=\frac{TP}{TP+ FP}\\ {} sen=\frac{TP}{TP+ FN}\\ {}F1=\frac{2\bullet pre\bullet sen}{pre+ sen}\end{array}\right. $$

where *TP*, *FP* and *FN* indicate the true positive (correctly identified vessel pixels), false positive (incorrectly identified vessel pixels) and false negative (incorrectly identified background pixels), respectively.

### Results

In our experiments, all the parameter settings are empirical. In detail, diameter of the circular structural element $d={d}^{\prime }=8.5/\left(2\ast p\right),{\lambda}_1={\lambda}_2={\lambda}_1^{\prime }={\lambda}_2^{\prime }=2.1/\max \left(M1,M2\right)$*, r* = *r*^′^ = 5, *p* = 0.3*, α*_1_ = *α*_2_ = 0.5*, M*1 and *M*2 where are the size in each dimension of an image.

**Fig. 2:**
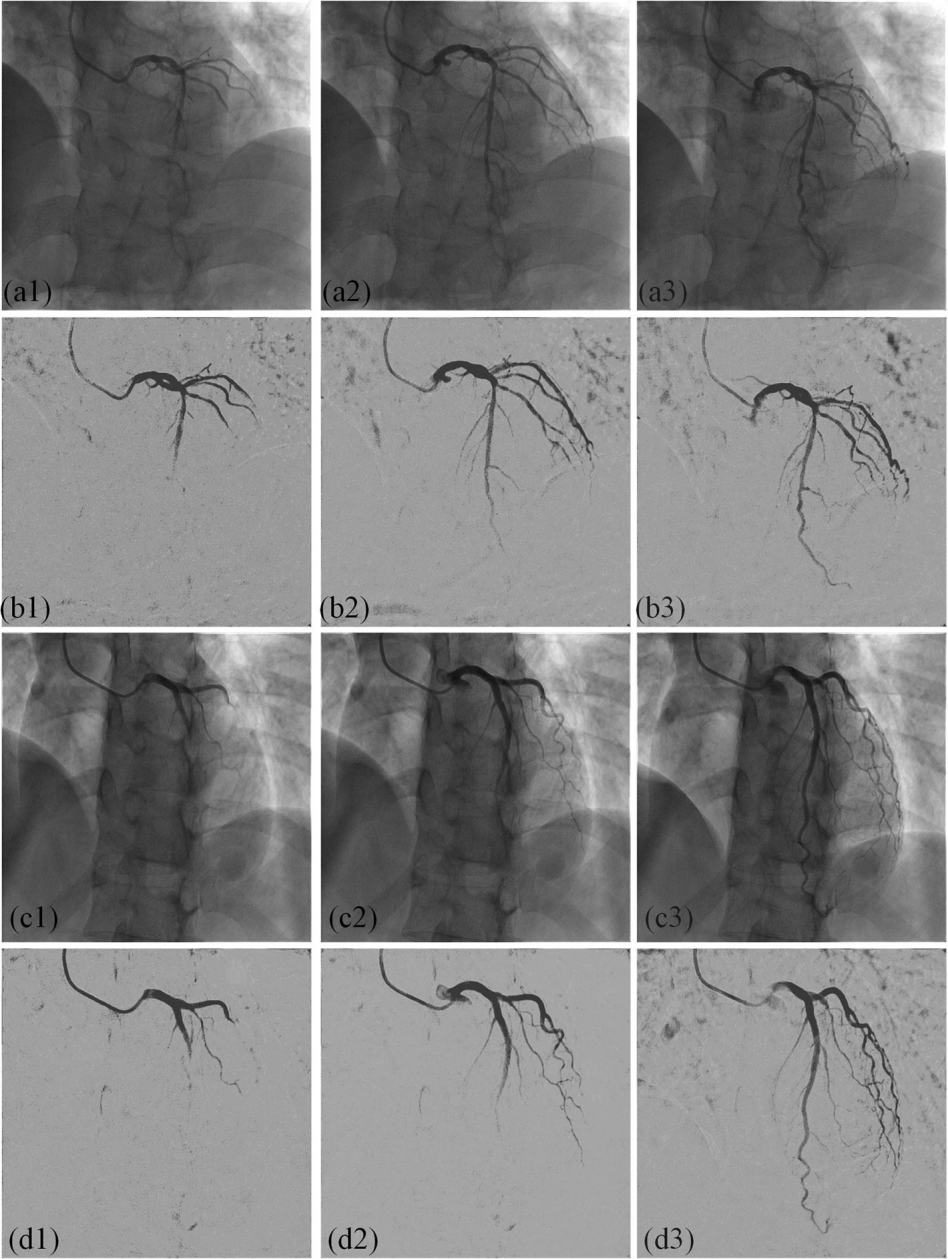
An example of extracted vascular images by the proposed method. (a1)-(a3), (c1)-(c3) original XRA images in two different sequences, (b1)-(b3), (d1)-(d3) extracted vascular images by IFC-RPCA method

Figure 2 shows the extracted vascular images by the proposed IFC-RPCA method. The order numbers of the three random selected images in the first two rows are *17th, 23th, 31th*. The order numbers of images in the last two rows are *19th, 24th and 41th*, respectively. According to the order, the inflow of contrast agent is gradually infused within the coronary artery. In Figs. 2(a1) and (c1), the contrast agent is not fully infused within the coronary artery. In Figs. 2(a2) and (c2), vessels are in the diastole stage, while in Figs. 2(a3) and (c3), vessels are in the systole stage. As can be seen from the extracted vascular images, the vascular structures are preserved throughout the XRA sequences and present a very high contrast. In addition, motion artefacts and other non-vascular noise are also removed and vascular segments with small scales are also preserved.

**Fig. 3:**
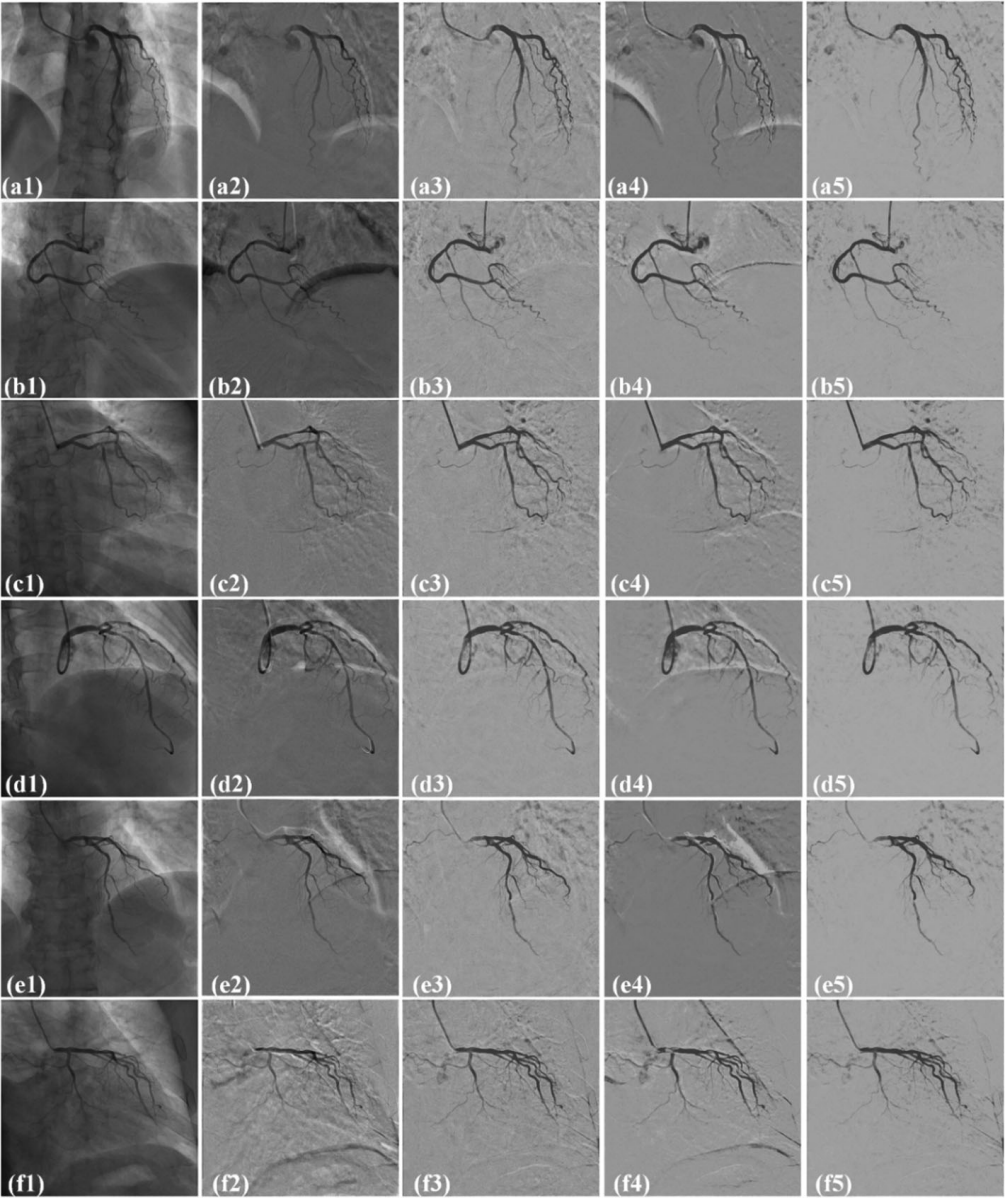
Comparison of the extracted vascular images on six randomly selected images from six different sequences by four methods. (a1-f1): original XRA images; (a2-f2), (a3-f3), (a4-f4) and (a5-f5): extracted vascular images by MER, ORPCA, MCR-RPCA and IFC-RPCA methods

Figure 3 shows the comparison results by four methods, including MER, ORPCA, MCR-PCA and IFC-RPCA, respectively. Six XRA images, as shown in Figs. 3(a1) to (f1), are randomly selected from six different sequences. In Figs. 3(a2)-(f2), MER introduces much motion artefacts near diaphragm, catheter and other large intensity variation regions. In Figs. 3(a3)-(f3), ORPCA removes much non-vascular noise, but introduces serious motion artefacts around the catheter and diaphragm. In Figs. 3(a4)-(f4), MCR-RPCA produces strong artefacts near the vessels, diaphragm, and lung boundaries. Images in Figs. 3(a5)-(f5) are computed by the proposed IFC-RPCA method. Artefacts caused by the catheter, lung tissues, diaphragm and vessels are almostly removed.

Table 1 compares the global CNR and local CNR over all the annotated XRA images by four different methods, including MER, ORPCA, MCR-RPCA and IFC-RPCA, respectively. All the methods obtain larger global CNRs than the original images and greatly improve the contrast of vessels. The global CNR of IFC-RPCA is much larger than MER and yields 17.37 and 37.91% improvement by comparing with ORPCA and MCR-RPCA, respectively. The performance of IFC-RPCA is achieved by removing the artefacts near the vessels, catheters and non-vascular noise. For the local CNR, the values by MER is smaller than the local CNR of original images which demonstrates that MER cannot improve the contrast within perivascular regions. IFC-RPCA obtains 10.20 and 47.46% improvement by comparing with ORPCA and MCR-RPCA, respectively. The proposed IFC-RPCA can also make the boundaries of the vascular structures much clearer.

**Table 1:** Comparison of local and global CNR by four different methods, including MER, ORPCA, MCR-RPCA and IFC-RPCA over 22 XRA images

Methods	Local CNR	Global CNR
Original Image	1.2175 ± 0.3838	0.8259 ± 0.2685
MER	0.1259 ± 0.0597	0.1709 ± 0.1143
ORPCA	3.8914 ± 0.5323	5.6527 ± 1.0719
MCR-RPCA	2.9081 ± 0.7021	4.8105 ± 1.3528
IFC-RPCA	4.2882 ± 0.7430	6.6344 ± 1.0849

**Fig. 4:**
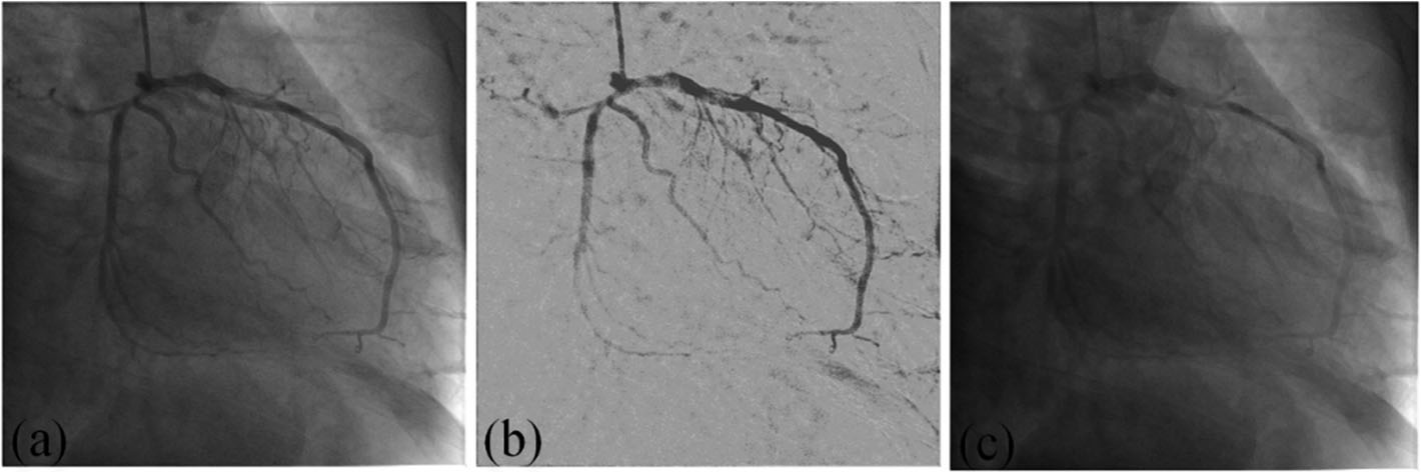
Simulated image with low dose contrast agent. (a) original image; (b) enhanced image; (c) simulated image

We also simulate the angiograms with low dose contrast agent which has significant clinical value for the clinicians and patients. The simulated images are generated by linearly subtracting the enhanced vascular image from the original image. Figure 4 shows an example of the simulated image with low dose contrast agent. As can be seen from Fig. 4(c), the contrast of the vessels is greatly reduced.

**Fig. 5:**
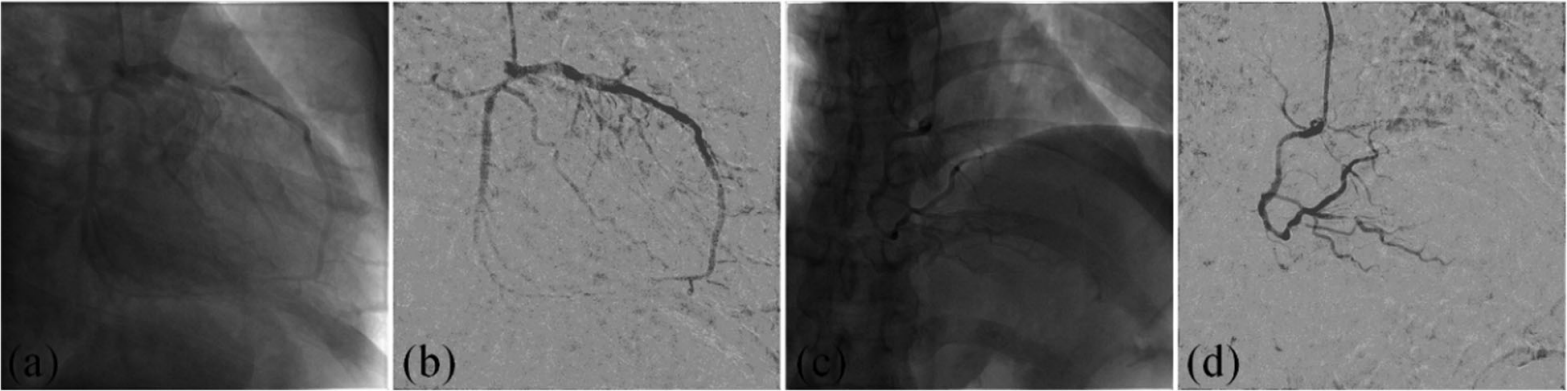
Enhanced results based on simulated images with low dose contrast agent. **a** and **c** simulated images; **b** and **d** enhanced results

Figure 5 shows the subtraction results based on the simulated images with low dose contrast agent. In Fig. 5a, it is very difficult to distinguish the vascular structures from the background. While in Fig. 5c, the intensity of the vessels is very close to the bones. As can be seen from Figs. 5b and d, the proposed IFC-RPCA method can improve the contrast of the vessels and meanwhile, remove the diaphragm and bones.

**Fig. 6:**
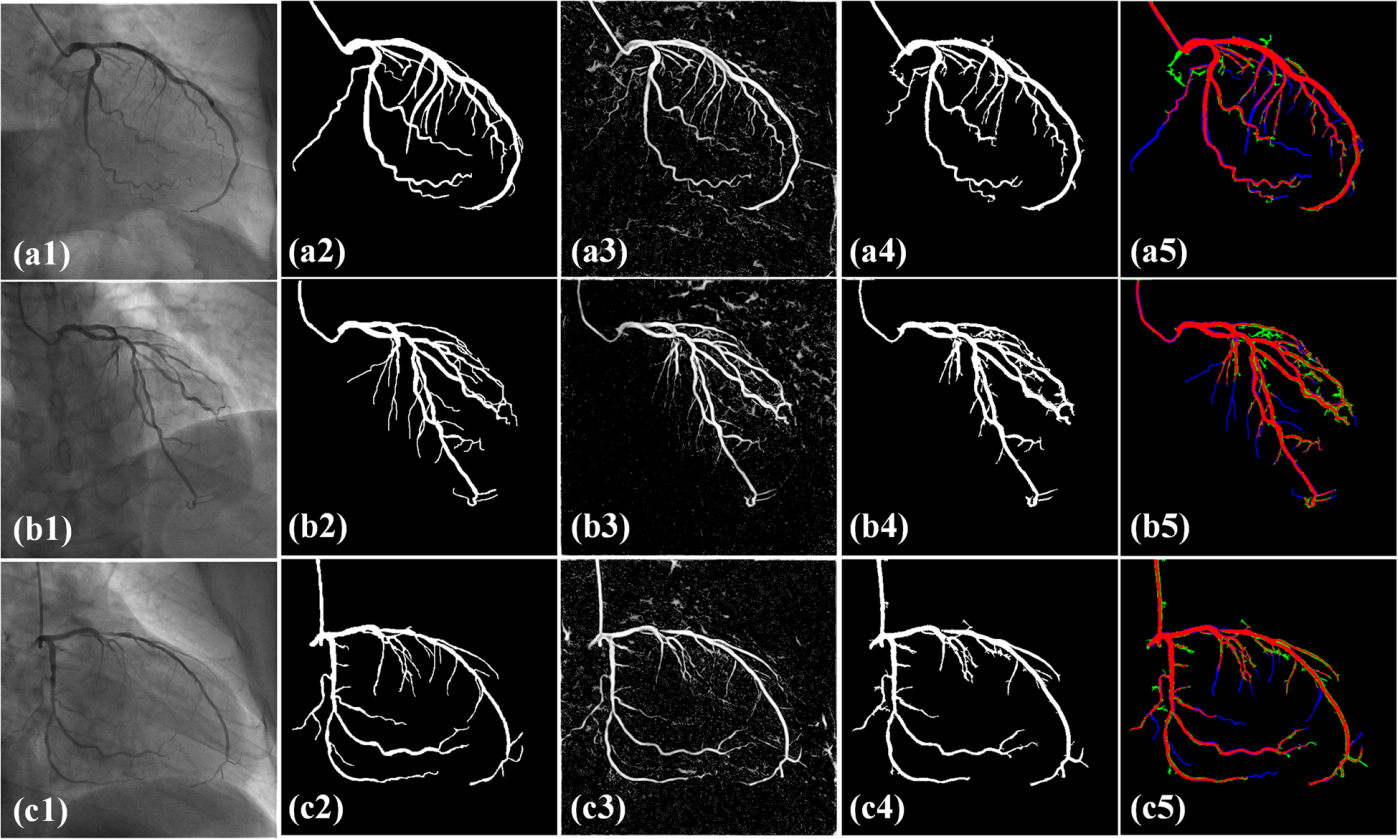
Segmentation results by the proposed method. (a1)-(c1) original angiograms; (a2)-(c2) ground truth; (a3)-(c3) Multi-feature fused restuls; (a4)-(c4) segmented results; (a5)-(c5) color map between the ground truth and segmented results. Red color: correctly identified vessel pixels, green color: incorrectly identified vessel pixels and blue color: incorrectly identified background pixels

Fig. 6 shows the segmented vascular structures by the proposed method. Angiograms in the first column are randomly selected from image sequences. Images in the third column are computed by the proposed feature descriptor. In the images, there are uniform responses when the vascular segments appear bifurcation, overlapping or are very close to each other. In addition, the responses in vascular regions are much larger than the background which brings the vessels high contrast. Images in the fourth column refer to the segmentation results by a threshold value from images in the third column. In the images, the vascular edges are preserved even when different vascular segments are very close. In addition, vascular segments with small scales are also accurately segmented. In the fifth column, vascular segments in green color refer to over-segmentation, while vascular segments in blue color refer to under-segmentation. As can be seen from the figures, the vessels will be fractured when the intensity of vascular segments is close to the background. For the vessels with large scales, they have precise boundaries and are consistent with the ground truth. As also can be seen from the segmented results, the non-vascular noise is almost removed.

**Fig. 7:**
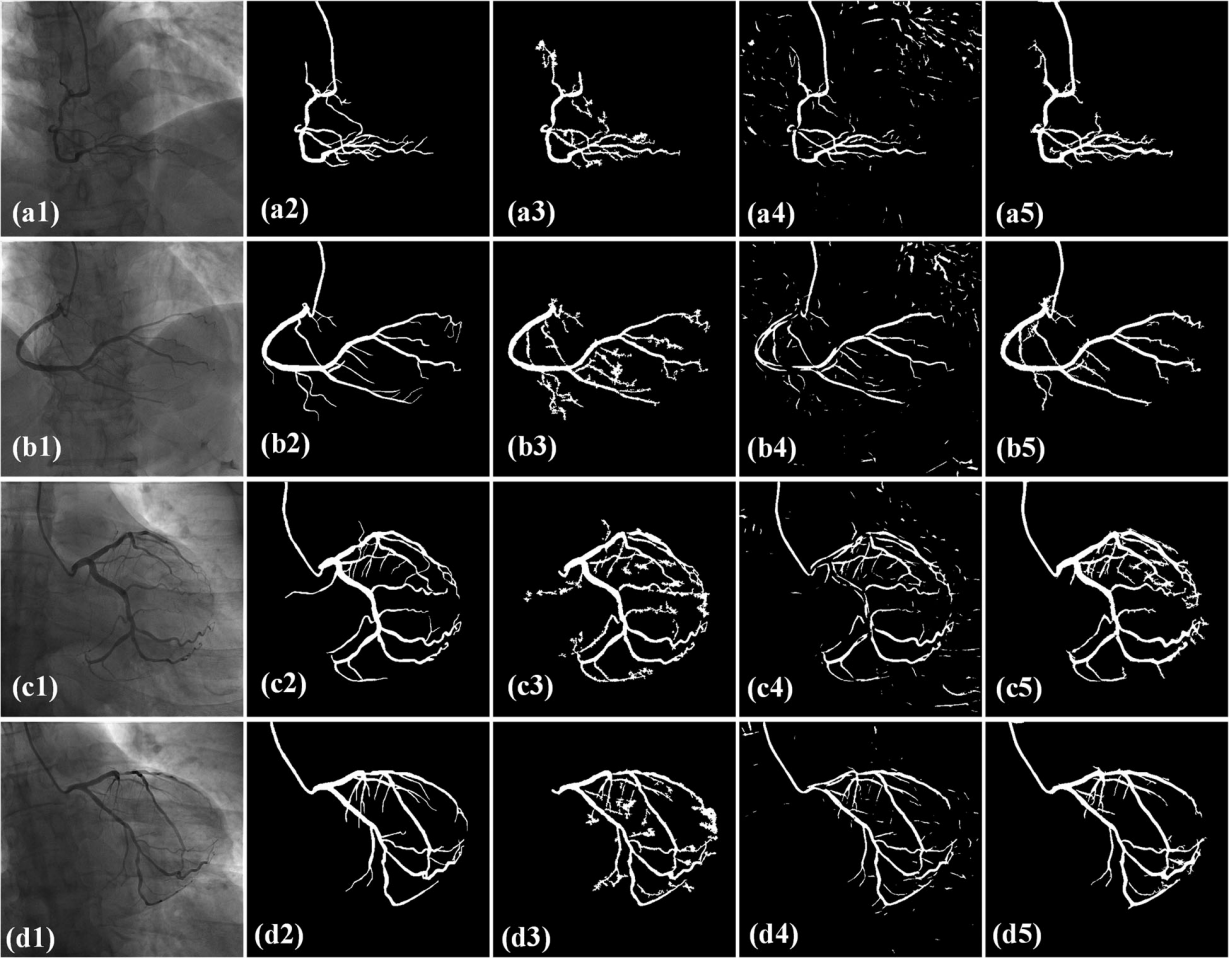
Qualitative comparison between different methods. (a1)-(d1) original angiograms; (a2)-(d2) ground truth; (a3)-(d3) segmented results by LevelSet; (a4)-(d4) segmented results by FC-CRF; (a5)-(d5) segmented results by the proposed method

Figure 7 shows the comparison results by methods, including LevelSet, FC-CRF and the proposed model. In the first column, two right and two left coronary artery angiograms are randomly selected from the image sequences. In the third column, vascular segments with large scales are precisely segmented and vascular segments with small scales present serious noise. In addition, LevelSet method is semi-automatic and requires the manual labeled seed points. In the fourth column, large amounts of noise appears in the non-vascular regions and there are many holes in the vascular segments. After comparing with the ground truth, the diameters of the obtained vascular segments are smaller than the actual vessels which make a great influence to the subsequent parameter measurement of vascular segments. In the fifth column, there are fractures when the vascular segments appear small-scales. But the vascular segments with large scales are precisely segmented. In addition, when the vascular segment is overlapped with the diaphragm, the segmentation results doesn’t introduce motion artefacts. For each angiogram in the figures, the segmentation results by the proposed method present a clean background without non-vascular noise. Table 2 provides the quantitative comparison in *pre, sen* and *F1* value by LevelSet, FC-CRF and the proposed method. *Pre, sen* and *F1* value of the proposed method are all the largest, the vessels and noise are both disposed best. All the metrics of LevelSet are all superior to those of FC-CRF. The vascular structure preservation and background noise removing are all better than these of FC-CRF.

**Table 2:** Quantitative segmenation comparison of the proposed method with CF-CRF and LevelSet

methods	*pre*	*sen*	*F1* value
LevelSet	0.7025	0.7430	0.7222
CF-CRF	0.6314	0.6875	0.6583
proposed method	0.7378	0.7960	0.7658

## Conclusion and discussion

In the paper, we propose an inter/intra-frame constrained vascular segmentation method and demonstrate its application in the XRA sequences. Experimental results demonstrate the effectiveness of the proposed method in accurate vessel segmentation in the XRA sequence. As can be seen from the experimental results, the proposed IFC-RPCA effectively reduces the lung tissues, diaphragm and vertebral bodies and removes the motion artefacts near the catheter and non-vascular noises from the XRA sequence. The proposed IFC-RPCA yields 17.37 and 37.79% improvement in global CNR and 10.20 and 47.46% improvement by comparing with ORPCA and MCR-RPCA methods. The proposed multi-feature fused feature descriptor produces uniform response in different vascular segments and makes the vascular structures high contrast with the background. Based on this, the vascular structures can be simply segmented with only a threshold value. We obtain 0.7378, 0.7960 and 0.7658 with respect to the precision, sensitivity and F1 value, respectively. It demonstrates the proposed method can both effectively dispose the vessels and background. The proposed vessel segmentation method is online without re-optimizing the whole model and automatic, it is very suitable to be applied in the intra-operative image guided surgical navigation.

## Data Availability

The data is not shared with outside institutions.

## Abbreviations

CAD: Coronary artery disease
CNN: Convolutional neural network
CNR: Contrast-to-noise ratio
CRF: Conditional random field
FC-CRF: Fully-connected conditional random field
FN: False negative
FP: False positive
IFC-RPCA: Inter/infra-frame constrained RPCA
LevelSet: Level-set-based method
MCR-RPCA: The graduated RPCA with motion coherency constraint
MER: Multiresolution elastic registration
ORPCA: Online robust principal component analysis
pre: Precision
RPCA: Robust principal component analysis
sen: Sensitivity
TP: True positive
XRA: X-ray angiography
